# The Biomechanical Signature of Elite Distance Running: A Matched Pairs Analysis Across Multiple Speeds

**DOI:** 10.1111/sms.70385

**Published:** 2026-10-21

**Authors:** C. Mayo‐Nieto, A. Cartón‐Llorente, A. Rafales‐Perucha, S. Cardiel‐Sánchez, D. Jaén‐Carrillo, L. E. Roche‐Seruendo

**Affiliations:** ^1^ Universidad San Jorge Zaragoza Spain; ^2^ Department of Sport Science University of Innsbruck Innsbruck Austria

**Keywords:** foot kinematics, ground reaction force, leg stiffness, running biomechanics, running economy, spatiotemporal parameters, spring‐mass model, wearable sensors

## Abstract

This study compared the biomechanical profiles of elite and recreational distance runners across five measurement domains using wearable inertial sensors across treadmill speeds of 10–15 km/h. A total of 104 runners (52 elite; 52 recreational) were assessed using RunScribe sensors during an incremental treadmill protocol. Twenty‐three variables spanning spatiotemporal parameters, stiffness and force, impact loading, foot kinematics and vertical dynamics were recorded at each speed. Elite runners were matched 1:1 with recreational runners by sex, age, height, and body mass. For each variable, a linear mixed‐effects model (LMM) was fitted to within‐pair differences with pair‐level random intercepts and speed slopes, and speed and within‐pair body mass and age imbalances as fixed effects. FDR correction (Benjamini–Hochberg) was applied to the 23 group‐effect and 23 interaction tests independently. Twelve of 23 variables (52.2%) showed a significant group effect, and six (26.1%) a significant group×speed interaction after FDR correction. Elite runners showed shorter contact time (*d*_z_ = −0.73 to −0.53), higher flight ratio (*d*_z_ = +0.52 to +0.66) and a more anterior footstrike pattern at initial contact (*d*_z_ = +0.58 to +0.67). RunScribe‐estimated vertical GRF rate and leg stiffness were also higher than in recreational runners (*d*_z_ = +0.48 to +0.75, and + 0.51 to +0.62, respectively). Step frequency and step length showed no overall group difference. Significant group×speed interactions revealed that, for most affected variables, values changed more steeply with speed in recreational runners. These findings describe a distinct mechanical profile in elite runners across the six evaluated submaximal speeds.

## 1 Introduction

Understanding the determinants of distance running performance is a central question in sports sciences. Historically, distance running performance has been explained through a physiological lens; the dominance of East African runners has directed research toward altitude adaptation, anthropometric profile, dietary habits, and ethnic characteristics as the principal determinants of elite success [1]. More recently, the interest in marginal gains from biomechanics has become highly visible with the emergence and subsequent popularization of carbon‐fiber plate running shoes [2], which have demonstrated significant improvements in running efficiency [3]. Indeed, when groups of distance runners with equivalent VO_2_max are compared, running economy and biomechanical efficiency have emerged as key differentiating factors of performance [4].

To understand this biomechanical efficiency, numerous studies have sought to differentiate elite from recreational runners through the analysis of kinematic and spatiotemporal variables at controlled speeds. With regard to stride mechanics, it has been reported that high performance runners tend to adopt greater step lengths and lower step frequencies than their recreational counterparts [5], while others report comparable stride length with a longer flight time instead [6]. A lower contact time (in %) has also been reported in expert compared with novice runners at controlled speeds (10 and 15 km/h) [7]. Concerning spring‐mass properties, elite athletes have been shown to run as stiffer mechanical systems, exhibiting greater vertical and leg stiffness across the full speed spectrum [8], with their kinetics more closely resembling an idealized elastic spring‐mass template [9]. Differences have also been reported in ground reaction force characteristics between performance levels, including a lower vertical loading rate and a greater vertical impulse in faster runners [10]. Moreover, regarding foot kinematics, elite athletes have further been reported to predominantly adopt a forefoot strike pattern, contacting the ground closer to their centre of mass (CoM) and with a larger ankle moment, compared to recreational runners [6], although this has not been consistently replicated [11].

Traditionally, the measurement of kinematic variables has been restricted to complex and costly motion capture systems or force platforms confined to laboratory settings. However, the advent of wearable devices has made it possible to evaluate running mechanics in real‐world environments [12]. Foot‐mounted inertial measurement units (IMUs), such as the commercial RunScribe sensor, provide an extensive array of metrics spanning multiple biomechanical domains relevant to running performance: spatiotemporal parameters, stiffness and force, impact and shock, foot kinematics, gait phase durations and vertical dynamics. Nonetheless, the validity of these metrics against reference systems has not been uniformly established across all domains. While basic spatiotemporal variables have shown good agreement [13, 14], evidence is discordant or limited for impact, force and stiffness metrics [15, 16, 17], and mixed for foot kinematics [13, 15, 17]. Given this variable‐dependent level of validation across such a broad metric set, a detailed appraisal of the validity of the reported RunScribe metrics is provided in Section 2.

Despite this analytical potential, the existing literature on elite‐recreational biomechanical comparisons is constrained by three critical limitations. First, most studies have employed small sample sizes, frequently *n* < 10–22 per group, limiting statistical power and the generalizability of findings [12]. Second, comparisons have typically focused on a narrow set of variables, predominantly spatiotemporal parameters, leaving entire biomechanical domains, including foot kinematics and impact loading profiles, largely unexplored in performance‐level comparisons. Third, few studies have adequately controlled for anthropometric confounders considering that body mass, height, and sex exert substantial influences on biomechanical parameters [18], and elite runners typically differ from recreational runners in such characteristics. Simple group comparisons without anthropometric matching risk conflating genuine performance‐related adaptations with body size effects, potentially explaining the partially inconsistent findings reported across studies [19, 20, 21]. As a result, the biomechanical signature that truly distinguishes elite from recreational runners remains incompletely characterized.

The purposes of this study were therefore (1) to compare a comprehensive set of biomechanical variables across five measurement domains between elite and recreational distance runners at six standardized treadmill speeds (10–15 km/h), using an anthropometrically matched pairs design controlling for sex, age, height, and body mass; and (2) to identify which biomechanical domains and individual variables most consistently differentiate performance levels. We hypothesized that elite runners would exhibit a mechanical profile consistent with a stiffer spring‐mass interaction with the ground, characterized by shorter ground contact time, longer flight time, and greater leg stiffness compared to recreational runners.

## 2 Methods

### 2.1 Participants

A total of 172 runners (55 elite [ELT], 44M/11F, and 117 recreational [CON], 82M/35F) voluntarily participated in the study. Following matching and outlier removal, 52 matched ELT–CON pairs were retained for analysis (see Matched‐Pairs Design and Quality Control). Inclusion criteria for the ELT group required a documented 10 km personal best under 33 min (males) or under 36 min (females) achieved within the 12 months prior to testing. CON runners were required to run at least 3 days per week with a weekly volume exceeding 25 km, and to have no high‐competitive‐level background. Exclusion criteria for both groups included any musculoskeletal injury that prevented normal running for more than seven consecutive days during the 3 months prior to the study, any major lower‐limb surgery, and being under 18 years old. Participants wore their usual running shoes to enhance ecological validity; however, the use of minimalist shoes or carbon‐fiber plate reinforced shoes was not permitted to avoid mechanical interference. This study complied with the ethical standards of the World Medical Association's Declaration of Helsinki (2013). All participants provided written informed consent, and the study was approved by the regional ethics committee (CEICA, Aragón, Spain; C.I. PI24/133). Participants were informed of their right to withdraw from the study at any time.

### 2.2 Instrumentation and Protocol

Biomechanical data were collected using the RunScribe (Scribe Labs Inc., Half Moon Bay, CA) inertial measurement units, sampling at 500 Hz, attached bilaterally to the shoelaces during an incremental treadmill protocol on a motorized treadmill (WOODWAY Pro XL, Woodway Inc., Waukesha, WI, USA) set at 0% gradient. Following a 6‐min warm‐up at a comfortable self‐selected speed, participants completed the incremental protocol in a fixed ascending order from 10 to 15 km/h, 1 min per stage, following manufacturer calibration procedures performed before each session. Rest between stages was not mandatory but was permitted on request to minimize the influence of accumulated fatigue and isolate the biomechanical component of interest. The first 30 s of each stage were discarded as an accommodation period, and data from the final 30 s of each stage were retained for analysis; this window exceeds the minimum duration (10 s, 25–30 steps) previously reported as sufficient for reliable assessment of spatiotemporal running variables at comparable submaximal treadmill speeds [22] and follows a recording design consistent with prior RunScribe‐based protocols using the same treadmill model [23]. Testing was conducted in a temperature‐ and humidity‐controlled laboratory (20°C–22°C, 45%–55% relative humidity). Left and right limb values were averaged for bilateral variables. Twenty‐three variables were extracted at each speed, organized into five measurement domains (Table 1).

**TABLE 1:** Biomechanical variables extracted from the RunScribe foot‐mounted IMU system, organized by measurement domain.

Domain—Variable	Definition
Spatiotemporal	
Step frequency (SF)	The number of steps per minute (steps/min)
Step length (SL)	Distance from the initial contact of one foot to the initial contact of the contralateral foot (m)
Contact time (CT)	Duration from initial contact to toe‐off (ms)
Flight time (FT)	Duration of the period during which neither foot is in contact with the ground (ms)
Contact ratio (Cratio)^b^	Percentage of the total step duration spent in contact with the ground (%)
Flight ratio (Fratio)^b^	Percentage of the total step duration during which both feet are in the air (%)
Stride angle (SA)^b^	The angle of the foot's velocity vector relative to the horizontal ground plane at the instant of toe‐off (i.e., the take‐off trajectory angle) (°) [24, 25]
Stiffness & Force	
Leg stiffness (Kleg)^a^	Ratio of maximum ground reaction force (*F*_max_) to the displacement of Centre of Mass (CoM) in the direction of the line between the CoM and the point of force application during contact (kN/m)
Vertical stiffness (Kvert)^a^	Ratio of *F*_max_ to the vertical displacement of the CoM during the ground contact phase (kN/m)
Peak vertical GRF^a^	Estimate of the peak vertical ground reaction force at mid‐stance, normalized by body weight
Vertical GRF rate^a^	Rate of vertical ground reaction force development during the stance phase, normalized to body weight (BW/s)
Horizontal GRF rate^a^	Rate of horizontal ground reaction force development during the propulsive phase of stance, normalized to body weight (BW/s)
Total force rate^**a**^	Weighted sum of the vertical/horizontal components of the GRF rate and the rate of force required to swing the limb, normalized to body weight (BW/s)
Impact & Shock	
Shock (Gs)	Composite metric combining Impact and Braking Gs, representing total shock per footstep (g)
Braking Gs	Horizontal component of Peak Gs; correlates with the braking forces at footstrike (g)
Impact Gs	Vertical component of Peak Gs; correlates with the ground impact force at footstrike (g)
Medio‐lateral Gs^b^	Side‐to‐side peak accelerations at footstrike (g)
Foot kinematics	
Footstrike type (FST)	Ordinal index (1–16) reflecting foot position relative to the ground in the sagittal plane at initial contact: from 1 (rearfoot), to 16 (forefoot) [25]
Pronation excursion	Total range of inward angular movement (roll) from footstrike to maximum pronation (°)
Max. pronation velocity	Maximum angular rate at which the foot pronates between footstrike and maximum pronation (°/s)
Swing excursion^b^	Difference in pitch between the furthest rearward and forward portions of the swing (°)
Vertical dynamics	
Vertical oscillation^b^	Total vertical displacement of the CoM during the gait cycle (cm)
Vertical speed^b^	Vertical velocity of the shoe at the moment of footstrike (m/s)

^a^ RunScribe‐estimated: computed by proprietary theoretical algorithms, without criterion validation in running.

^b^ Sensor‐derived: no criterion validation in running.

Validity evidence for RunScribe outputs during running differs by outcome. Basic spatiotemporal variables, such as contact time and cycle time, showed excellent consistency with 3D motion capture (ICC: 0.91–0.94), although contact time was overestimated by about 10% [13]. Against high‐speed video, contact time, flight time, step frequency, and step length showed ICCs of 0.85–0.97, with small systematic differences and the smallest bias for lace‐mounted sensors [14]. For footstrike, categories correlated with foot angle at initial contact with lace‐mounted sensors (|*r*| = 0.86; overall accuracy 78%, 55% for midfoot) [15], but the 1–16 footstrike index was not correlated with foot strike angle with heel‐mounted sensors (*r* = 0.09) [17]. For pronation, maximum pronation velocity showed good consistency with 3D motion capture (ICC: 0.74–0.87) but was overestimated by up to 150°/s, and pronation excursion showed fair consistency (ICC: 0.40–0.57) and was underestimated by up to 4° [13]. Impact and shock accelerations showed low to moderate associations with vertical loading rate (*r* = 0.55 and 0.47) [16]. GRF estimates (peak vertical GRF and GRF rates) are computed by proprietary algorithms, and leg and vertical stiffness with a spring‐mass model applied to IMU‐derived data. No criterion validation of these outputs in running was found, so they are referred to as RunScribe‐estimated throughout the text. By the same rule, the remaining outputs without criterion validation (stride angle, contact and flight ratios, medio‐lateral Gs, swing and stance kinematics, phase durations, vertical oscillation, and vertical speed) are referred to as sensor‐derived. The pelvis kinematics module did not reach the validity criteria for any variable or speed tested [23] and was excluded from the analysis. Similarly, gait phase duration variables (stance and swing sub‐phases) were excluded, as their computation relies on the accurate identification of intermediate events by proprietary algorithms, for which no criterion validity evidence during running was identified.

### 2.3 Matched‐Pairs Design

Each ELT runner was matched 1:1 with a CON runner using the following procedure: (a) exact matching on sex; (b) within each sex stratum, standardized Euclidean distance was computed on age, height, and body mass (each variable divided by its pooled standard deviation); (c) a greedy nearest‐neighbor algorithm without replacement was used, processing harder‐to‐match subjects first. This yielded 52 matched pairs (41 male, 11 female).

### 2.4 Quality Control: Outlier Detection and Management

A systematic outlier detection protocol was applied before analysis. For each variable at each speed, modified Z‐scores based on the Median Absolute Deviation (MAD) were computed within each group separately [26]. Subjects with |modified *Z*| > 3.5 on ≥ 5% of their available variable‐speed combinations were classified as problematic outliers. Three Tier 1 ELT subjects (> 11% outlier values, including values with |*Z*| > 40 indicating likely sensor artifacts) were identified and their pairs removed. Seven CON subjects flagged as outliers were replaced using the same nearest‐neighbor matching procedure.

Following subject‐level screening, individual data points were cleaned in two stages. Stage 1 removed values outside pre‐specified plausibility limits for variables with defined physical bounds (step frequency: 120–300 spm, contact time: 100–500 ms, flight time: 0–200 ms, contact ratios: 0%–100%, footstrike type: 1–16) [18], which identified likely sensor artifacts. Stage 2 applied a modified *Z*‐score threshold (|MAD‐*Z*| > 3.5) computed within each group‐speed cell, removing statistical outliers not captured by the physiological limits. In total, 156 observations of 13 721 available data points (1.12%) were removed (11 in Stage 1 and 145 in Stage 2). Only pairs with valid data for both members at a given variable and speed entered the LMM, representing 97.77% of the available data (Table S1).

### 2.5 Statistical Analysis

For each of the 23 biomechanical variables, a linear mixed‐effects model (LMM) was fitted to the within‐pair differences (ELT minus CON) across the six running speeds. The model was: Δ*y* = *β*_0_ + *β*_1_ × speed_c + *β*_2_ × Δmass + *β*_3_ × Δage + *b*_0,pair_ + *b*_1,pair_ × speed_c + *ε*, where speed_c is running speed centered at the midpoint of the tested range (12.5 km/h), Δmass and Δage are the residual within‐pair imbalances in body mass and age, and *b*_0,pair_ and *b*_1,pair_ are pair‐level random intercepts and random slopes for speed, capturing the correlation among repeated measurements within each pair and allowing the ELT–CON difference to change with speed differently across pairs. An observation was included in each variable's model only when both members of a pair had valid data at that speed; pairs with missing data at some speeds were retained for the variables and speeds available. Models were fitted by restricted maximum likelihood (REML) using the lme4 (version 2.0.6) and lmerTest (version 3.2.1) packages in R (version 4.6.1), with Satterthwaite approximation for degrees of freedom. The intercept (*β*_0_) estimates the mean ELT–CON difference at the midpoint speed; the *β*_1_ coefficient tests whether the group difference changes linearly across the speed range (group × speed interaction). Body mass was preferred over BMI as a covariate given its direct mechanical relationship with ground reaction forces; a model with BMI is reported as sensitivity analysis. Effect sizes were computed separately at each running speed as Cohen's *d*_z_ (mean within‐pair difference at the speed divided by its standard deviation). The 95% confidence interval for each *d*_z_ was derived from the noncentral *t* distribution. Effect sizes were interpreted as small (*d*_z_ = 0.20–0.49), medium (*d*_z_ = 0.50–0.79), or large (*d*_z_ ≥ 0.80) for descriptive purposes, acknowledging that *d*_z_ is not directly comparable to between group *d* [27]. Benjamini–Hochberg FDR correction was applied across the 23 group‐effect *p*‐values and, separately, across the 23 interaction *p*‐values. LMM coefficients are reported as *β*_0_ [95% CI].

A priori power analysis was based on a within‐pair comparison equivalent to the LMM group effect. In the absence of previous studies using a matched‐pairs design, the contact time difference reported by Preece et al. [6] between high performance and recreational runners (*d* = 0.44) was used as a proxy for the within‐pair effect size; a minimum of 43 matched pairs was required to achieve 80% power. The primary analysis sample of 45–52 pairs met or exceeded this threshold across all speeds. The minimum detectable effect size with 80% power ranged from *d*_z_ = 0.40 (*n* = 52) to *d*_z_ = 0.43 (*n* = 45), indicating that the study was adequately powered to detect medium and large effects but may have lacked sensitivity for effects below *d*_z_≈0.40.

Two sensitivity analyses were conducted to assess the robustness of the primary LMM results. First, replacing body mass with BMI as a covariate produced concordant FDR decisions for 21 of 23 group effects and all 23 interaction effects; swing excursion and vertical speed did not retain a significant group effect. Second, given the ordinal nature of footstrike type (1–16 scale), within‐pair differences for this variable were re‐tested using the Wilcoxon signed‐rank test with Benjamini–Hochberg FDR correction; results were consistent with the LMM inference at all six speeds (*q* ≤ 0.001).

During the preparation of this work the authors used Claude (Anthropic, Opus 5.5) in order to assist with statistical code review and debugging, and with checking the proper use of the English language. After using this service, the authors reviewed and edited the content as needed and took full responsibility for the content of the published article.

## 3 Results

### 3.1 Matching Quality

After matching and quality control, 52 ELT‐CON pairs were retained. Sex was perfectly balanced by design, and height showed negligible residual imbalance (ELT: 175.8 ± 8.4 vs. CON: 175.2 ± 8.4 cm, SMD = +0.07, *p* = 0.229). Meaningful residual within‐pair differences persisted for body mass (ELT: 64.7 ± 9.2 vs. CON: 67.1 ± 8.9 kg, SMD = −0.27, *p* < 0.001), age (ELT: 27.3 ± 6.4 vs. CON: 31.6 ± 10.7 year, SMD = −0.49, *p* = 0.002), and BMI (ELT: 20.8 ± 1.8 vs. CON: 21.8 ± 1.8, SMD = −0.53, *p* < 0.001). These differences reflect the inherently younger age profile and leaner body composition of competitive distance runners rather than a failure of the matching procedure. Within‐pair differences in body mass and age were included as covariates in the LMM to partially account for residual anthropometric imbalance, though statistical adjustment does not render the groups anthropometrically equivalent. Standardized mean differences before and after matching for all variables are reported in Table S2.

### 3.2 Sample Availability Across Speeds

The number of available matched pairs decreased modestly across the 10–15 km/h range: 52 pairs at 10 km/h (100%), 50 at 11–12 km/h (96%), 49 at 13 km/h (94%), 47 at 14 km/h (90%), and 45 at 15 km/h (87%). This speed range was selected to maintain adequate statistical power (≥ 45 pairs at all speeds) while avoiding the severe attrition observed at higher velocities. The demographic profile of available pairs remained stable across speeds: mean ELT age ranged from 29 to 31 years, CON age from 33 to 35 years, ELT body mass from 64 to 66 kg, and CON body mass from 67 to 69 kg. This indicates that while sample size decreased, the anthropometric balance of the matched pairs was preserved at all speeds. Point‐level quality control (Table S1) subsequently removed a small number of additional observations from specific variable‐speed‐group cells, reducing the minimum available pairs for any single variable from 45 to 41 (median 48 across all variable‐speed combinations).

### 3.3 Overview of Biomechanical Differences

Of 23 LMM group‐effect tests (one per variable), 12 (52.2%) revealed a significant ELT–CON difference after FDR correction. Six variables (26.1%) additionally showed a significant group×speed interaction, indicating that the magnitude of the ELT–CON difference changed systematically with running speed. The distribution of significant group effects across domains was: spatiotemporal (5/7, 71.4%), stiffness and force (4/6, 66.7%), foot kinematics (2/4, 50.0%), vertical dynamics (1/2, 50%), and zero for impact/shock (0/4). Figure 1 presents the heatmap of Cohen's *d*_z_ effect sizes across all variables and speeds. The complete LMM results, including group‐effect estimates, 95% confidence intervals, and group × speed interaction tests for all 23 variables, are presented in Table 2. Group means ± SDs for all variables at each running speed are provided in Table S3.

**FIGURE 1:**
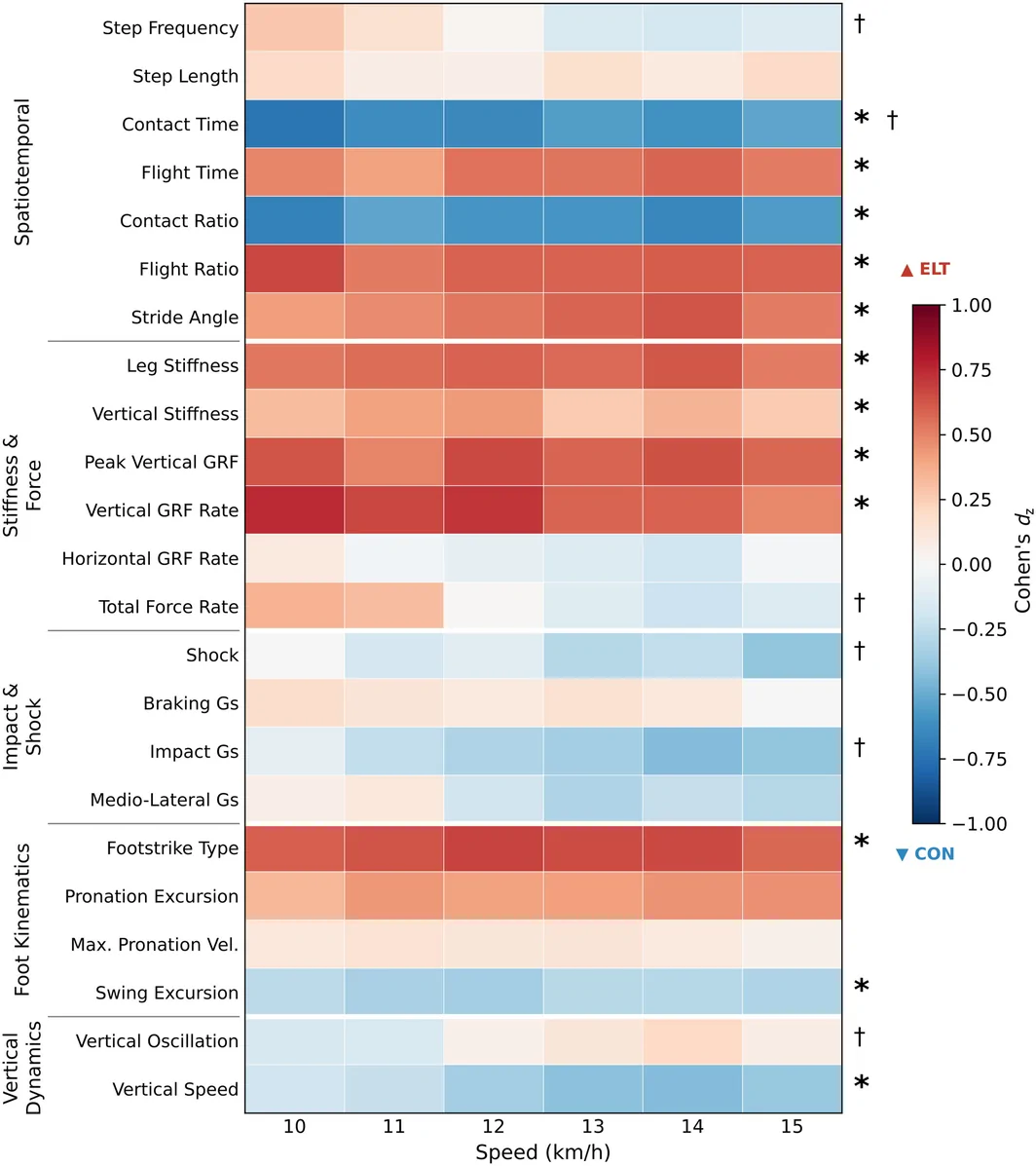
Heatmap of Cohen's *d*_z_. effect sizes for 23 biomechanical variables across six running speeds (10–15 km/h), organized by measurement domain. Positive values (red) indicate elite runners scored higher than recreational counterparts; negative values (blue) indicate the reverse. Color intensity reflects effect magnitude. Asterisks indicate a statistically significant group effect in the linear mixed‐effects model after FDR correction (Benjamini–Hochberg; one test per variable). Daggers indicate a significant group‐by‐speed interaction.

**TABLE 2:** Linear mixed‐effects model results for all 23 biomechanical variables.

Domain—Variable	*β*₀	95% CI	*p*	*q* (FDR)	*d*_z_	*p* (int.)	*q* (int.)
Spatiotemporal							
Step frequency (steps/min)	+0.83	[−2.16, +3.82]	0.579	0.701	−0.17 to +0.27	< 0.001	0.001**
Step length (m)	+0.02	[−0.02, +0.05]	0.379	0.485	+0.07 to +0.19	0.947	0.962
Contact time (ms)	−14.04	[−22.10, −5.98]	0.001	0.008**	−0.73 to −0.53	0.002	0.008**
Flight time (ms)	+12.38	[+2.45, +22.30]	0.016	0.033*	+0.41 to +0.58	0.820	0.944
Contact ratio	−3.65	[−6.13, −1.17]	0.005	0.017*	−0.67 to −0.52	0.233	0.357
Flight ratio	+3.54	[+1.08, +6.01]	0.006	0.017*	+0.52 to +0.66	0.296	0.425
Stride angle (deg)	+0.57	[+0.14, +0.99]	0.010	0.025*	+0.42 to +0.63	0.962	0.962
Stiffness & force							
Leg stiffness (kN/m)	+0.83	[+0.31, +1.36]	0.003	0.012*	+0.51 to +0.62	0.405	0.548
Vert. stiffness (kN/m)	+0.76	[+0.24, +1.28]	0.005	0.017*	+0.26 to +0.43	0.833	0.944
Peak vGRF (BW)	+0.29	[+0.11, +0.47]	0.003	0.012*	+0.49 to +0.66	0.811	0.944
vGRF rate (BW/s)	+2.12	[+1.04, +3.21]	< 0.001	0.004**	+0.48 to +0.75	0.170	0.301
hGRF rate (BW/s)	−0.01	[−0.21, +0.18]	0.885	0.911	−0.20 to +0.10	0.082	0.171
Total force rate (BW/s)	+1.24	[−1.03, +3.50]	0.277	0.374	−0.21 to +0.35	0.001	0.008**
Impact & shock							
Shock (g)	−0.38	[−1.06, +0.29]	0.262	0.374	−0.39 to +0.01	0.002	0.008**
Braking Gs (g)	+0.03	[−0.47, +0.53]	0.911	0.911	−0.01 to +0.17	0.059	0.135
Impact Gs (g)	−0.63	[−1.35, +0.08]	0.081	0.144	−0.43 to −0.10	0.002	0.009**
Medio‐lateral Gs (g)	−0.08	[−0.18, +0.02]	0.128	0.210	−0.30 to +0.11	0.023	0.058
Foot kinematics							
Footstrike type (a.u.)	+2.87	[+1.36, +4.39]	< 0.001	0.004**	+0.58 to +0.67	0.862	0.944
Pronation excursion (deg)	+1.35	[−0.78, +3.49]	0.209	0.321	+0.33 to +0.46	0.017	0.057
Max pronation Vel (deg/s)	−4.56	[−84.06, +74.93]	0.909	0.911	+0.05 to +0.14	0.226	0.357
Swing excursion (deg)	−5.97	[−10.50, −1.45]	0.011	0.025*	−0.35 to −0.26	0.159	0.301
Vertical dynamics							
Vertical oscillation (cm)	−0.12	[−0.69, +0.44]	0.667	0.767	−0.16 to +0.20	0.001	0.008**
Vertical speed (m/s)	−0.20	[−0.36, −0.03]	0.018	0.035*	−0.43 to −0.19	0.021	0.058

*Note:* FDR significance: *q < 0.05, **q < 0.01, ***q < 0.001. Speed‐specific sample sizes: 10 km/h *n* = 52, 11 km/h *n* = 50, 12 km/h *n* = 50, 13 km/h *n* = 49, 14 km/h *n* = 47, 15 km/h *n* = 45.

Abbreviations: CI, 95% confidence interval; *d*_z_, Cohen's *d*_z_ (within‐pair standardized mean difference; range reflects min and max values across 10–15 km/h); int., group‐by‐speed interaction; *p*, unadjusted *p*‐value; *q*, Benjamini–Hochberg FDR *p*‐value; *β* **₀**, group‐effect estimate at 12.5 km/h.

### 3.4 Spatiotemporal Parameters

ELT runners showed shorter contact time (*d*_z_ = −0.73 to −0.53) and a lower contact ratio (*d*_z_ = −0.67 to −0.52), with a complementary increase in flight ratio (*d*_z_ = +0.52 to +0.66), consistent with a more pronounced aerial phase. Stride angle was greater in ELT across all speeds (*d*_z_ = +0.42 to +0.63). No overall group difference was found for step frequency or step length; however, step frequency showed a significant group × speed interaction: cadence increased with speed in both groups but rose more steeply in recreational runners, indicating that ELT runners maintain a more stable cadence profile as speed increases.

### 3.5 Stiffness and Force Application

The stiffness and force domain showed consistent group differences. ELT runners showed higher estimated vertical GRF loading rates (*d*_z_ = +0.48 to +0.75) and peak vertical GRF (*d*_z_ = +0.49 to +0.66), and greater RunScribe‐estimated leg stiffness (*d*_z_ = +0.51 to +0.62) and vertical stiffness (*d*_z_ = +0.26 to +0.43). Horizontal and total GRF rates showed no significant group main effect but total force rate showed a significant group × speed interactions, indicating that group divergence in these force characteristics was speed‐dependent.

### 3.6 Impact Loading and Shock

No impact variable reached FDR significance for the overall group effect. However, shock and impact showed significant group × speed interactions (*q* = 0.008 and *q* = 0.009, respectively), suggesting that differences in axial impact accelerations between ELT and CON runners increased progressively across the speed range tested rather than being uniformly present.

### 3.7 Foot Kinematics

ELT runners exhibited a more anterior footstrike pattern (*d*_z_ = +0.58 to +0.67) and lower swing excursion than CON runners, although the latter had small effect sizes (*d*_z_ = −0.35 to −0.26).

### 3.8 Vertical Dynamics

Vertical oscillation showed no overall group effect but a significant group×speed interaction. In contrast, vertical speed was more negative in ELT than CON runners (*d*_z_ = −0.43 to −0.19) indicating a faster downward foot velocity at landing, with no significant group×speed interaction. This group effect was not retained in the BMI‐adjusted sensitivity analysis.

Figure 2 illustrates the speed‐dependent trajectories of key biomechanical variables for elite and recreational level runners across 6 running speeds (10–15 km/h).

**FIGURE 2:**
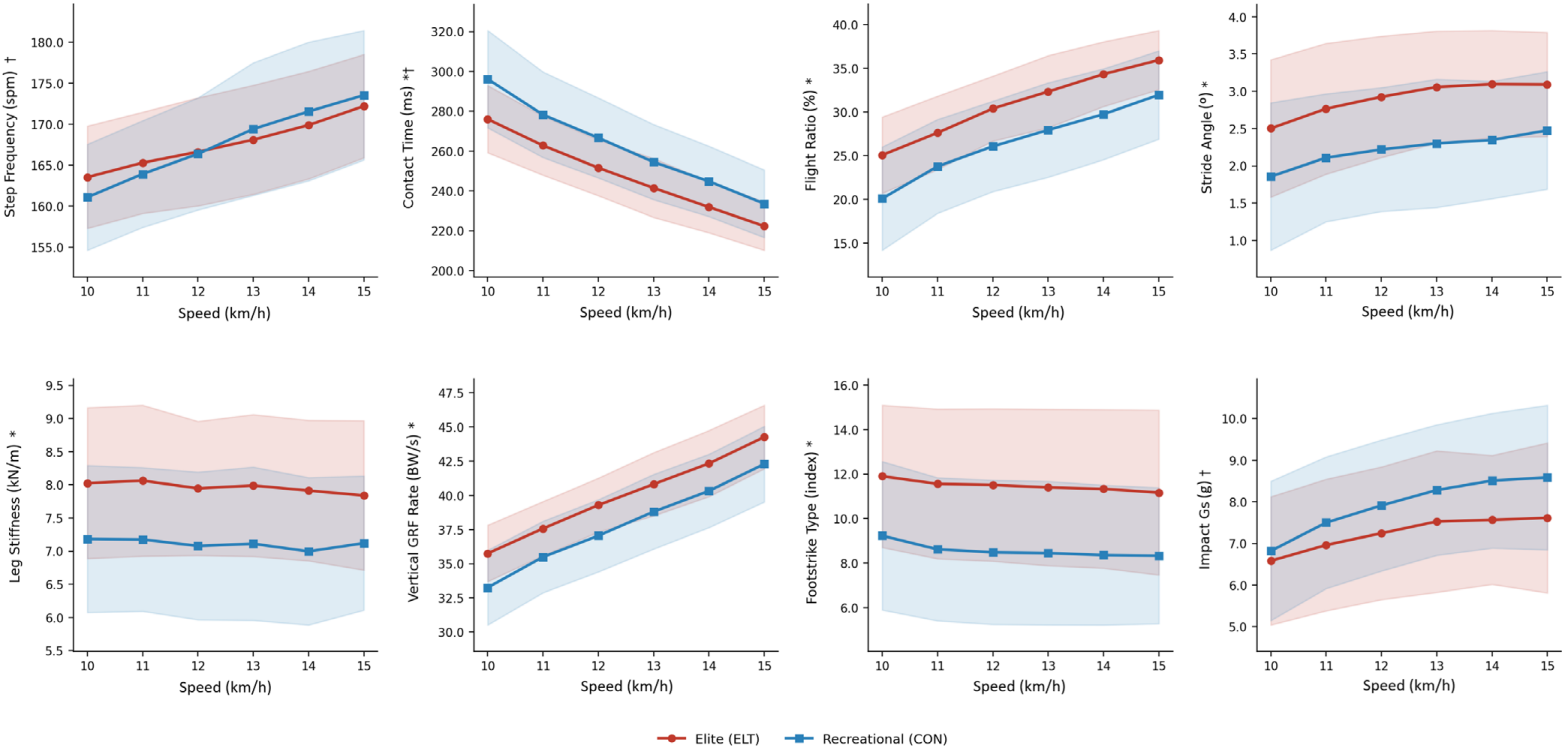
Mean trajectories across running speeds for eight key variables in elite (red) and recreational (blue) matched pairs. Shaded bands represent ±1 SD of between‐subject variability. Asterisks indicate a significant group main effect. Daggers indicate a significant group × speed interaction.

## 4 Discussion

This study provides the first large‐scale, anthropometrically‐matched, multi‐domain biomechanical comparison between elite and recreational distance runners. By analyzing 23 variables across five measurement domains at six speeds, a coherent biomechanical signature of elite running that extends well beyond conventional spatiotemporal parameters was identified. Five principal findings emerged: First, elite runners showed shorter ground contact time and longer flight time, both in absolute and relative values. Second, elite runners exhibited a more anterior footstrike pattern. Third, elite runners showed a distinct force application profile characterized by greater RunScribe‐estimated leg stiffness and vertical ground reaction force loading rate. Fourth, step frequency and step length showed no significant group main effect. Fifth, significant group × speed interactions in 6 of 23 variables indicate that the magnitude of the ELT–CON difference was speed‐dependent for a limited subset of the evaluated biomechanical parameters. We interpret this convergent pattern in ELT runners as consistent with a stiffer and more elastic interaction with the ground, although metabolic cost, elastic energy return, and muscle‐tendon behavior were not measured directly in this study, and this interpretation should therefore be regarded as a hypothesis rather than a directly demonstrated mechanism.

### 4.1 A Multi‐Domain Spring‐Mass Signature

The convergent pattern across domains showing shorter contact time, greater flight time, and leg stiffness, faster vertical GRF loading rate, and a more anterior footstrike pattern describes a coordinated biomechanical strategy consistent with a stiffer, more elastic interaction with the ground. This pattern aligns with the spring‐mass model prediction that more efficient runners should exhibit greater “spring‐mass similarity” [9], a bounce that more closely resembles the idealized elastic template.

The greater leg stiffness (*d*_z_ = +0.51 to +0.62) aligns with Burns et al. [8], who reported +2.1 kN/m greater leg stiffness and +3.6 kN/m greater vertical stiffness in sub‐four‐minute milers compared to highly trained counterparts, and with Zhang et al. [28], who found that, compared to recreational runners, elite athletes applied greater GRF in both vertical and anterior–posterior directions (*d* = +1.63 to +2.03) with a longer aerial time (*d* = +1.11) at 12 km/h. These findings contrast with García‐Pinillos et al. [5], who found no leg stiffness differences between 10 elite and 12 novice runners, a discrepancy likely reflecting the absence of anthropometric matching, the smaller sample size, or differences in participant definitions and measurement methods in that study. When body mass, a primary determinant of stiffness [18, 29], is controlled, performance‐related stiffness differences may become more detectable. Van Hooren et al. [30] confirmed an association between leg stiffness and running economy (*r* = −0.28), supporting the functional relevance of this finding, further corroborated by a meta‐analysis [31] and a narrative review [32] showing that both vertical and leg stiffness correlate negatively with running economy.

The faster RunScribe‐estimated vertical GRF loading rate in elite runners (*d*_z_ = +0.48 to +0.75) contrasts with Evans et al. [10], who reported a higher loading rate in 27 recreational compared to 35 collegiate runners during overground force plate running (194.5 vs. 111.5 BW/s). This comparison, however, was made at self‐selected rather than matched speeds, with collegiate runners running substantially faster than recreational runners (4.1 vs. 3.5 m/s); Evans et al. attributed their finding primarily to footstrike pattern, noting that the more anterior footstrike of their collegiate runners likely offset the loading‐rate increase otherwise expected from their higher speed, and explicitly identified speed‐matched comparisons as a direction for future work. Notably, our elite runners also showed a more anterior footstrike pattern than recreational runners (*d*_z_ = +0.58 to +0.67), yet this coexisted with a higher, not lower, estimated vertical loading rate at matched absolute speeds, suggesting that footstrike pattern alone does not account for the loading‐rate differences observed here. Together with the greater RunScribe‐estimated leg stiffness, this faster loading profile suggests that elite runners apply force more explosively, within a shorter time window, a pattern that may reflect superior musculotendinous stiffness and elastic energy storage capacity, although these mechanisms were not directly measured. Rapid muscle‐tendon loading during early stance is hypothesized to potentiate elastic recoil during push off, and the coordinated stiffness‐loading profile observed here is consistent with this stretch‐shortening cycle framework, which has been linked to reduced metabolic cost of force production in prior experimental work [33, 34].

The shorter contact time (*d*_z_ = −0.73 to −0.53) and lower contact ratio (−0.67 to −0.52) in elite runners align with the lower duty factor reported in expert runners at 10 and 15 km/h by Fadillioglu et al. [7], but contrast with the nonsignificant differences reported by Preece et al. [6] (*d* = −0.44, *p* = 0.25) in 14 high performance vs. 14 recreational runners at 11.9–20.2 km/h, and García‐Pinillos et al. [5], who found no contact time differences across 10–18 km/h in 10 elite and 12 novice runners. The emergence of medium‐to‐large effect sizes in our matched‐pairs design suggests that body mass and height confounders may have obscured these differences in prior unmatched comparisons. Functionally, this shorter contact time, combined with the faster RunScribe‐estimated vertical GRF loading rate, is compatible with the self‐optimization framework proposed by Moore et al. [35], who demonstrated that contact time and leg stiffness are jointly tuned to minimize metabolic cost, with most runners preferring contact times shorter than the mathematical optimum, favoring elastic energy storage over prolonged force application, optimizing, therefore, the use of the stretch‐shortening cycle.

### 4.2 Foot Kinematics: Footstrike Pattern and Mechanical Correlates

Among foot kinematic variables, only footstrike type showed a significant group main effect, with ELT runners exhibiting a consistently more anterior initial contact pattern across all tested speeds (*d*_z_ = +0.58 to +0.67). This is consistent with Preece et al. [6], who reported a predominantly forefoot strike in 14 high performance runners vs. a rearfoot pattern in 14 recreational runners at controlled overground speeds (footstrike index 56% vs. 30% of foot length, *d* = 1.17). Race‐based observations have similarly documented a higher prevalence of non‐rearfoot striking among faster finishers [36], but this field study is methodologically limited for performance‐level comparisons, as faster runners inevitably run at higher absolute speeds and footstrike pattern is strongly speed‐dependent. The absence of a significant group×speed interaction suggests that this difference reflects an ingrained technical adaptation rather than a consequence of running speed. The more anterior footstrike is mechanically coherent with the broader set of group differences identified here. A more distal initial contact point has been associated with lower duty factor and a stiffer, more extended lower limb [37]. Together these features are compatible with the spring‐mass signature described above [38].

Critically, the absence of an overall group difference in pronation excursion indicates that the foot kinematic signature of elite runners is not about “over‐pronation” control but about the efficiency of the entire stance phase. Notably, ELT runners also showed lower swing excursion (*d*_z_ = −0.35 to −0.26). Combined with their more anterior footstrike, this more compact swing pattern may reflect an active ground search before initial contact. However, this effect was small and not retained in the BMI‐adjusted sensitivity analysis. A joint‐level evaluation using 3D motion capture combined with principal component analysis could help confirm or refute this interpretation.

### 4.3 Quality Over Quantity: The Stride Cycle Redistribution

Perhaps the most striking finding across the entire analysis was the absence of overall group differences in step frequency and step length. These are the two primary mechanical levers for increasing running speed: a runner can go faster by taking more steps or longer steps. Yet on average across the tested speeds, anthropometrically matched ELT and CON runners did not differ in either lever. However, the LMM revealed a significant group×speed interaction for step frequency (FDR *q* = 0.001), indicating that the two groups diverge in their cadence response to increasing speed: recreational runners increased step frequency more steeply across the tested range (+12.4 steps/min from 10 to 15 km/h) compared to elite runners (+8.7 steps/min). This suggests that at higher speeds, elite runners rely proportionally less on cadence increments and more on other mechanisms, presumably longer flight time, to increase velocity. Burns et al. [9] partially support this interpretation, as they reported that elite runners show less spatiotemporal variability across speeds.

These results are in line with Preece et al. [6], who reported comparable stride length between high performance and recreational runners, achieved via a prolonged aerial phase rather than greater knee extension at landing, but contrasts with García‐Pinillos et al. [5], who found longer step length and lower step frequency in ELT runners. The discrepancy may reflect the anthropometric matching used in this study: unmatched ELT runners may appear to have longer steps simply because they are taller or lighter, a confound previously reported in analyses of the determinants of running parameters [18]. When confounders are controlled, the “how” of each step, not the “how many” or “how long”, appears to differentiate performance levels. Elite runners redistribute their stride cycle duration toward less ground contact and more flight time, achieving higher RunScribe‐estimated peak forces and faster vertical GRF loading rates within that shorter contact window.

Stride angle, however, was significantly greater in ELT runners across speeds (*d*_z_ = +0.42 to +0.63), indicating that elite runners achieve a relatively higher flight arc per unit of step length. Importantly, this did not translate into greater vertical oscillation of the CoM, which showed no overall difference. Within the impact domain, no variable reached FDR significance for the overall group effect; instead, shock and impact Gs exhibited significant group×speed interactions, with estimated vertical impact accelerations rising more steeply with speed in recreational runners. In addition, vertical speed at foot contact was more negative in ELT runners (*d*_z_ = −0.43 to −0.19), indicating a faster downward foot velocity at landing. Taken together with the greater RunScribe‐estimated stiffness observed in ELT runners, these findings suggest that ELT runners may achieve a longer aerial phase not by oscillating more vertically overall, but by redistributing this oscillation within the stride: greater stiffness and shorter contact time, implying less CoM lowering during stance, and greater stride angle and flight time, implying a relatively higher CoM trajectory during the aerial phase. However, this pattern is also compatible with the greater relative physiological demand that standardized absolute speeds impose on recreational runners, whose mechanical responses may therefore reflect reduced elastic buffering capacity at higher intensities. Whether this divergence persists when speeds are matched by physiological markers rather than absolute velocity remains an open question that warrants investigation in future studies, given its relevance from both a performance and a running‐related injury risk perspective, although the current evidence linking discrete impact metrics to running‐related injury remains only low‐to‐moderate, and causal interpretations should be made cautiously [39].

For coaches and practitioners, these results carry direct practical implications. The elite biomechanical signature identified here: greater leg stiffness, faster vertical loading, shorter contact time, and a more anterior footstrike is consistent with greater musculotendinous stiffness. Experimental evidence shows that plyometric training increases musculotendinous stiffness and improves distance running performance [33] and running economy [34]. Although this study did not directly test whether such interventions could modify the biomechanical characteristics observed here, this biomechanical signature may represent a plausible target for future intervention research. From a monitoring perspective, contact ratio, defined as the proportion of stride time spent in ground contact and analogous to duty factor, emerges as one of the most informative wearable‐accessible metrics, showing one of the largest and most consistent group effects in the analysis (*d*_z_ = −0.67 to −0.52). This variable has previously been shown to reliably classify running styles into aerial and terrestrial patterns [40], and its speed‐independence in the present dataset makes it particularly suitable for longitudinal monitoring of training‐induced adaptations. Stride angle can also provide a practical composite indicator of aerial phase efficiency that integrates both contact time and flight height into a single metric [24]. Together, these variables offer coaches and practitioners a focused, technology‐accessible toolkit for monitoring training‐induced adaptations in running mechanics.

Several limitations should be acknowledged. First, all variables were derived from RunScribe foot‐mounted IMUs rather than gold standard force plates or optical motion capture, which may affect stiffness and GRF estimates. Most validation studies used earlier device versions and, in some cases, heel placement, whereas sensors were attached to the shoelaces in the present study. Because both groups were measured with the same device, placement, and protocol, any systematic bias should affect ELT and CON runners similarly; however, absolute values should not be compared directly with laboratory‐derived measurements [14]. Second, the cross‐sectional design precludes causal inference. Third, the speed range was limited to 10–15 km/h to maintain adequate sample sizes (≥ 45 pairs) at all speeds; consequently, these findings may not generalize to velocities above 15 km/h where biomechanical patterns may differ further. In addition, within‐pair correlations were low, indicating that anthropometric matching controlled for confounding but did not reduce between‐pair variability. Thus, the a priori power estimate may have been optimistic for small effects, although the main group differences (*d*_z_ = 0.5–0.7) exceeded the minimum detectable effect size. Fourth, testing was conducted at matched absolute speeds rather than matched relative physiological intensities. Because elite and recreational runners differ substantially in aerobic capacity, the same speed represents a lower demand for ELT runners, which may partly explain the diverging mechanical responses observed at higher speeds. This remains a relevant direction for future research considering speed‐matching by relative intensity. Fifth, training load data were not collected in this study and therefore cannot be confirmed as balanced across matched pairs. Sixth, participants wore their habitual running shoes, which, while improving ecological validity, introduces a potential confound, as shoe properties may differ systematically between elite and recreational runners. Finally, an exploratory sex‐stratified analysis revealed consistent results in male pairs (*n* = 41), where 10 of 23 variables reached FDR significance with the same directional pattern as the primary analysis. In female pairs (*n* = 11), no variable survived FDR correction, although effect sizes remained directionally consistent with those observed in males, suggesting insufficient statistical power rather than a genuine absence of group effects. Future studies should prioritize the recruitment of larger female elite–recreational cohorts to characterize sex‐specific biomechanical profiles with adequate precision.

## 5 Perspective

Several directions emerge from these findings that merit future investigation. A first priority concerns the choice of testing condition: comparing groups at identical absolute speeds, as done here and in most existing literature, conflates mechanical and physiological demands, since the same velocity represents a very different relative effort for an elite and a recreational runner. Examining whether the biomechanical differences reported here persist or attenuate when groups are matched at physiologically equivalent intensities would provide a cleaner picture of the mechanics of fatigue resistance rather than absolute locomotor capacity. Beyond testing conditions, the biomechanical differences identified between performance levels are likely underpinned by structural and neuromuscular factors not assessed here, including tendon stiffness, muscle architecture, and neural drive, and future work should examine the extent to which these individual traits explain the between‐group differences in leg stiffness, contact ratio, and footstrike. Finally, contact ratio, measurable in ecological settings via foot‐mounted IMUs, warrants validation as a practical screening tool across competitive levels, given its demonstrated ability to classify running styles [40] and its robustness across speeds shown here. From a methodological standpoint, the 23 variables analysed here constitute a high‐dimensional biomechanical space in which individual variables may not capture the full covariance structure of the elite‐recreational contrast. Multivariate approaches such as principal component analysis could be applied to identify latent biomechanical dimensions that summarize group differences more parsimoniously and represent a natural extension of the variable‐by‐variable framework presented here.

## Funding

This work was supported by the Ayudas predoctorales Fundación Banco Sabadell ‐ Universidad San Jorge.

## Ethics Statement

The study was approved by the regional ethics committee (CEICA, Aragón, Spain; C.I. PI24/133).

## Consent

All participants provided written informed consent.

## Conflicts of Interest

The authors declare no conflicts of interest.

## Supporting information


**Table S1:** Summary of data quality control applied to each of the 23 RunScribe variables.

**Table S2:** Anthropometric characteristics of elite (ELT) and recreational (CON) runners before and after 1:1 nearest‐neighbor matching.

**Table S3:** Descriptive statistics and within‐pair effect sizes for elite (ELT) and recreational (CON) runners across six running speeds (10–15 km/h) for all 23 biomechanical variables included in the primary analysis.

## Data Availability

The data that support the findings of this study are available from the corresponding author upon reasonable request.

## References

[1] N. Tawa and Q. Louw , “Biomechanical Factors Associated With Running Economy and Performance of Elite Kenyan Distance Runners: A Systematic Review,” Journal of Bodywork and Movement Therapies 22, no. 1 (2018): 1–10, 10.1016/j.jbmt.2017.11.004. 29332730

[2] S. Lin , Y. Song , X. Cen , K. Bálint , G. Fekete , and D. Sun , “The Implications of Sports Biomechanics Studies on the Research and Development of Running Shoes: A Systematic Review,” Bioengineering 9, no. 10 (2022): 497, 10.3390/bioengineering9100497. PMC9598645 36290465

[3] Y. Xiao , X. Hu , D. Tian , and A. Qiu , “Effects of Advanced Footwear Technology on Running Economy and Endurance Performance: A Meta‐Analysis,” International Journal of Sports Medicine 47, no. 2 (2026): 81–94, 10.1055/a-2637-7283. 40527489

[4] P. U. Saunders , D. B. Pyne , R. D. Telford , and J. A. Hawley , “Factors Affecting Running Economy in Trained Distance Runners,” Sports Medicine 34, no. 7 (2004): 465–485, 10.2165/00007256-200434070-00005. 15233599

[5] F. García‐Pinillos , A. García‐Ramos , R. Ramírez‐Campillo , P. A. Latorre‐Román , and L. E. Roche‐Seruendo , “How Do Spatiotemporal Parameters and Lower‐Body Stiffness Change With Increased Running Velocity? A Comparison Between Novice and Elite Level Runners,” Journal of Human Kinetics 70 (2019): 25–38, 10.2478/hukin-2019-0036. PMC6942482 31915473

[6] S. J. Preece , C. Bramah , and D. Mason , “The Biomechanical Characteristics of High‐Performance Endurance Running,” European Journal of Sport Science 19, no. 6 (2019): 784–792, 10.1080/17461391.2018.1554707. 30556482

[7] C. Fadillioglu , F. Möhler , M. Reuter , and T. Stein , “Changes in Key Biomechanical Parameters According to the Expertise Level in Runners at Different Running Speeds,” Bioengineering 9, no. 11 (2022): 616, 10.3390/bioengineering9110616. PMC9687194 36354527

[8] G. T. Burns , R. González , J. M. Zendler , and R. F. Zernicke , “Bouncing Behavior of Sub‐Four Minute Milers,” Scientific Reports 11 (2021): 10501, 10.1038/s41598-021-89858-1. PMC8131362 34006954

[9] G. T. Burns , N. Tam , J. Santos‐Concejero , R. Tucker , and R. F. Zernicke , “Assessing Spring‐Mass Similarity in Elite and Recreational Runners,” Frontiers in Physiology 14 (2023): 1224459, 10.3389/fphys.2023.1224459. PMC10502723 37719459

[10] R. J. Evans , L. Herrington , and P. Comfort , “Injury and Performance Related Biomechanical Differences Between Recreational and Collegiate Runners,” Frontiers in Sports and Active Living 5 (2023): 1127749, 10.3389/fspor.2023.1127749. PMC10536965 37780121

[11] J. Gámez‐Payá , A. R. Aladro‐Gonzalvo , D. Gallego‐de Marcos , C. Villarón‐Casales , and J. L. Lopez‐del Amo , “Footstrike Pattern and Cadence of the Marathon Athletes at the Tokyo 2020 Olympic Games,” Applied Sciences 13, no. 11 (2023): 6620, 10.3390/app13116620.

[12] R. Mason , L. T. Pearson , G. Barry , et al., “Wearables for Running Gait Analysis: A Systematic Review,” Sports Medicine 53, no. 1 (2023): 241–268, 10.1007/s40279-022-01760-6. PMC9807497 36242762

[13] R. M. Koldenhoven and J. Hertel , “Validation of a Wearable Sensor for Measuring Running Biomechanics,” Digital Biomarkers 2, no. 2 (2018): 74–78, 10.1159/000491645. PMC7015375 32095758

[14] F. García‐Pinillos , J. M. Chicano‐Gutiérrez , E. J. Ruiz‐Malagón , and L. E. Roche‐Seruendo , “Influence of RunScribe Placement on the Accuracy of Spatiotemporal Gait Characteristics During Running,” Proceedings of the Institution of Mechanical Engineers, Part P: Journal of Sports Engineering and Technology 234, no. 1 (2020): 11–18, 10.1177/1754337119876513.

[15] A. F. DeJong and J. Hertel , “Validation of Foot‐Strike Assessment Using Wearable Sensors During Running,” Journal of Athletic Training 55, no. 12 (2020): 1307–1310, 10.4085/1062-6050-0520.19. PMC7740057 33064800

[16] C. Napier , R. W. Willy , B. C. Hannigan , R. McCann , and C. Menon , “The Effect of Footwear, Running Speed, and Location on the Validity of Two Commercially Available Inertial Measurement Units During Running,” Frontiers in Sports and Active Living 3 (2021): 643385, 10.3389/fspor.2021.643385. PMC8107270 33981991

[17] B. Pairot de Fontenay , J. S. Roy , B. Dubois , L. Bouyer , and J. F. Esculier , “Validating Commercial Wearable Sensors for Running Gait Parameters Estimation,” IEEE Sensors Journal 20 (2020): 7783–7791, 10.1109/JSEN.2020.2982568.

[18] L. Malisoux , C. Napier , P. Gette , N. Delattre , and D. Theisen , “Reference Values and Determinants of Spatiotemporal and Kinetic Variables in Recreational Runners,” Orthopaedic Journal of Sports Medicine 11, no. 10 (2023): 23259671231204629, 10.1177/23259671231204629. PMC10588426 37868213

[19] J. Gómez‐Molina , A. Ogueta‐Alday , J. Camara , C. Stickley , J. A. Rodríguez‐Marroyo , and J. García‐López , “Predictive Variables of Half‐Marathon Performance for Male Runners,” Journal of Sports Science and Medicine 16, no. 2 (2017): 187–194. PMC5465980 28630571

[20] A. Ogueta‐Alday , J. C. Morante , J. Gómez‐Molina , and J. García‐López , “Similarities and Differences Among Half‐Marathon Runners According to Their Performance Level,” PLoS One 13, no. 1 (2018): e0191688, 10.1371/journal.pone.0191688. PMC5783408 29364940

[21] J. Padulo , G. Annino , G. M. Migliaccio , S. D'Ottavio , and J. Tihanyi , “Kinematics of Running at Different Slopes and Speeds,” Journal of Strength and Conditioning Research 26, no. 5 (2012): 1331–1339, 10.1519/JSC.0b013e318231aafa. 22126973

[22] F. García‐Pinillos , P. Á. Latorre‐Román , R. Ramírez‐Campillo , J. A. Párraga‐Montilla , and L. E. Roche‐Seruendo , “Minimum Time Required for Assessing Step Variability During Running at Submaximal Velocities,” Journal of Biomechanics 80 (2018): 186–195, 10.1016/j.jbiomech.2018.09.005. 30243499

[23] E. J. Ruiz‐Malagón , F. García‐Pinillos , A. Molina‐Molina , V. M. Soto‐Hermoso , and S. A. Ruiz‐Alias , “RunScribe Sacral Gait Lab Validation for Measuring Pelvic Kinematics During Human Locomotion at Different Speeds,” Sensors 23, no. 5 (2023): 2604, 10.3390/s23052604. PMC10007442 36904808

[24] J. Santos‐Concejero , N. Tam , C. Granados , et al., “Interaction Effects of Stride Angle and Strike Pattern on Running Economy,” International Journal of Sports Medicine 35, no. 13 (2014): 1118–1123, 10.1055/s-0034-1372640. 24977947

[25] RunScribe , “RunScribe Pro Dashboard: Learn & Support [Stride Angle and Footstrike Type Metric Definitions]” (2026), Scribe Labs Inc. Registered Users Only.

[26] B. Iglewicz and D. C. Hoaglin , How to Detect and Handle Outliers (ASQC Quality Press, 1993).

[27] D. Lakens , “Calculating and Reporting Effect Sizes to Facilitate Cumulative Science: A Practical Primer for t‐Tests and ANOVAs,” Frontiers in Psychology 4 (2013): 863, 10.3389/fpsyg.2013.00863. PMC3840331 24324449

[28] J. H. Zhang , Z. Chan , F. O. Y. Lau , et al., “How Do Training Experience and Geographical Origin of a Runner Affect Running Biomechanics?,” Gait & Posture 84 (2021): 209–214, 10.1016/j.gaitpost.2020.12.003. 33360644

[29] R. Blickhan , “The Spring‐Mass Model for Running and Hopping,” Journal of Biomechanics 22, no. 11–12 (1989): 1217–1227, 10.1016/0021-9290(89)90224-8. 2625422

[30] B. Van Hooren , I. Jukic , M. Cox , K. G. Frenken , I. Bautista , and I. S. Moore , “The Relationship Between Running Biomechanics and Running Economy: A Systematic Review and Meta‐Analysis of Observational Studies,” Sports Medicine 54, no. 5 (2024): 1269–1316, 10.1007/s40279-024-01997-3. PMC11127892 38446400

[31] B. Liu , J. Wu , Q. Shi , et al., “Running Economy and Lower Extremity Stiffness in Endurance Runners: A Systematic Review and Meta‐Analysis,” Frontiers in Physiology 13 (2022): 1059221, 10.3389/fphys.2022.1059221. PMC9742541 36518102

[32] A. Struzik , K. Karamanidis , A. Lorimer , J. W. L. Keogh , and J. Gajewski , “Application of Leg, Vertical, and Joint Stiffness in Running Performance: A Literature Overview,” Applied Bionics and Biomechanics 2021 (2021): 9914278, 10.1155/2021/9914278. PMC8553457 34721664

[33] R. W. Spurrs , A. J. Murphy , and M. L. Watsford , “The Effect of Plyometric Training on Distance Running Performance,” European Journal of Applied Physiology 89, no. 1 (2003): 1–7, 10.1007/s00421-002-0741-y. 12627298

[34] C. Llanos‐Lagos , R. Ramírez‐Campillo , J. Moran , and E. Sáez de Villarreal , “Effect of Strength Training Programs in Middle‐ and Long‐Distance Runners' Economy at Different Running Speeds: A Systematic Review With Meta‐Analysis,” Sports Medicine 54, no. 4 (2024): 895–932, 10.1007/s40279-023-01978-y. PMC11052887 38165636

[35] I. S. Moore , K. J. Ashford , C. Cross , J. Hope , H. S. R. Jones , and M. McCarthy‐Ryan , “Humans Optimize Ground Contact Time and Leg Stiffness to Minimize the Metabolic Cost of Running,” Frontiers in Sports and Active Living 1 (2019): 53, 10.3389/fspor.2019.00053. PMC7739683 33344976

[36] H. Hasegawa , T. Yamauchi , and W. J. Kraemer , “Foot Strike Patterns of Runners at the 15‐Km Point During an Elite‐Level Half Marathon,” Journal of Strength and Conditioning Research 21, no. 3 (2007): 888–893, 10.1519/R-22096.1. 17685722

[37] A. Patoz , T. Lussiana , A. Thouvenot , L. Mourot , and C. Gindre , “Duty Factor Reflects Lower Limb Kinematics of Running,” Applied Sciences 10, no. 24 (2020): 8818, 10.3390/app10248818.

[38] I. S. Moore , “Is There an Economical Running Technique? A Review of Modifiable Biomechanical Factors Affecting Running Economy,” Sports Medicine 46, no. 6 (2016): 793–807, 10.1007/s40279-016-0474-4. PMC4887549 26816209

[39] A. Gruber , “The ‘Impacts Cause Injury’ Hypothesis: Running in Circles or Making New Strides?,” Journal of Biomechanics 156 (2023): 111694, 10.1016/j.jbiomech.2023.111694. 37364393

[40] A. Patoz , C. Gindre , A. Thouvenot , L. Mourot , K. Hébert‐Losier , and T. Lussiana , “Duty Factor Is a Viable Measure to Classify Spontaneous Running Forms,” Sports 7, no. 11 (2019): 233, 10.3390/sports7110233. PMC6915645 31717680

