# Supplementary material for: The Biomechanical Signature of Elite Distance Running: A Matched Pairs Analysis Across Multiple Speeds
Source: Scand J Med Sci Sports. 2026 Oct 21;36(11):e70385. doi: 10.1111/sms.70385 (PMC13687571; doi:10.1111/sms.70385)
Supplement: Supplementary file 1 [file SMS-36-e70385-s001.docx]

| Speed | 10 km/h | | 11 km/h | | 12 km/h | | 13 km/h | | 14 km/h | | 15 km/h | |
| --- | --- | --- | --- | --- | --- | --- | --- | --- | --- | --- | --- | --- |
| Variable | Removed ELT/CON | Valid pairs | Removed ELT/CON | Valid pairs | Removed ELT/CON | Valid pairs | Removed ELT/CON | Valid pairs | Removed ELT/CON | Valid pairs | Removed ELT/CON | Valid pairs |
| Step Frequency | 0/1 | 51 | 0/1 | 49 | 0/1 | 49 | 0/1 | 48 | 0/1 | 46 | 0/2 | 43 |
| Step Length | 1/1 | 50 | 1/1 | 48 | 2/2 | 46 | 1/1 | 47 | 1/1 | 45 | 1/0 | 44 |
| Contact Time | 1/1 | 50 | 0/0 | 50 | 0/0 | 50 | 0/0 | 49 | 1/0 | 46 | 1/0 | 44 |
| Flight Time | 0/1 | 51 | 2/1 | 47 | 1/1 | 48 | 1/0 | 48 | 1/0 | 46 | 2/1 | 43 |
| Contact Ratio | 2/0 | 50 | 0/0 | 50 | 0/0 | 50 | 0/0 | 49 | 1/0 | 46 | 1/1 | 43 |
| Flight Ratio | 2/0 | 50 | 0/0 | 50 | 0/0 | 50 | 0/0 | 49 | 0/0 | 47 | 1/0 | 44 |
| Stride Angle | 0/0 | 52 | 0/0 | 50 | 0/0 | 50 | 0/0 | 49 | 0/0 | 47 | 0/0 | 45 |
| Leg Stiffness | 0/1 | 51 | 1/0 | 49 | 1/0 | 49 | 1/0 | 48 | 1/0 | 46 | 1/1 | 43 |
| Vert. Stiffness | 2/3 | 47 | 2/1 | 47 | 0/1 | 49 | 0/1 | 48 | 0/2 | 45 | 0/1 | 44 |
| Peak vGRF | 1/0 | 51 | 3/1 | 46 | 1/1 | 48 | 1/0 | 48 | 1/0 | 47 | 2/1 | 43 |
| vGRF Rate | 0/0 | 52 | 1/0 | 49 | 0/0 | 50 | 0/0 | 49 | 0/0 | 47 | 0/0 | 45 |
| hGRF Rate | 2/3 | 47 | 1/3 | 46 | 1/3 | 46 | 1/1 | 47 | 1/3 | 44 | 1/4 | 41 |
| Total Force Rate | 2/1 | 49 | 1/1 | 48 | 1/1 | 49 | 2/1 | 46 | 2/2 | 43 | 1/0 | 44 |
| Shock | 0/0 | 52 | 1/0 | 49 | 0/2 | 48 | 1/0 | 48 | 0/0 | 47 | 1/1 | 43 |
| Impact Gs | 1/0 | 51 | 1/0 | 49 | 2/0 | 48 | 0/0 | 49 | 1/0 | 46 | 0/1 | 44 |
| Braking Gs | 0/0 | 52 | 0/0 | 50 | 0/1 | 49 | 0/0 | 49 | 0/0 | 47 | 0/1 | 44 |
| Mediolateral Gs | 0/0 | 52 | 1/1 | 48 | 0/0 | 50 | 0/0 | 49 | 1/2 | 44 | 1/0 | 44 |
| Footstrike Type | 0/0 | 52 | 0/0 | 50 | 0/0 | 50 | 0/0 | 49 | 0/0 | 47 | 0/0 | 45 |
| Pronation Excursion | 2/0 | 50 | 3/0 | 47 | 2/0 | 48 | 2/0 | 47 | 2/0 | 45 | 2/1 | 42 |
| Max Pronation Vel | 0/0 | 52 | 0/0 | 50 | 0/0 | 50 | 0/0 | 49 | 0/0 | 47 | 0/0 | 45 |
| Swing Excursion | 0/0 | 52 | 0/0 | 50 | 0/1 | 49 | 0/0 | 49 | 0/0 | 47 | 0/0 | 45 |
| Vertical Oscillation | 0/1 | 51 | 0/1 | 49 | 0/0 | 50 | 0/0 | 49 | 0/0 | 47 | 0/0 | 45 |
| Vertical Speed | 1/1 | 50 | 1/0 | 49 | 0/0 | 50 | 0/0 | 49 | 0/0 | 47 | 1/0 | 44 |
| Total | 17/14 | 97.4% | 19/11 | 97.4% | 11/14 | 97.9% | 10/5 | 98.7% | 13/11 | 98.0% | 16/15 | 97.3% |

Table S1. Summary of data quality control applied to each of the 23 RunScribe variables.

ELT: elite runners; CON = recreational runners; GRF = ground reaction force; MAD-Z = modified Z-score based on the median absolute deviation. Removed: observations excluded by quality control (QC) in each group (ELT/CON). Valid pairs: ELT-CON pairs with valid data for both members. Stage 1 removed physiologically implausible values for variables with defined physical bounds. Stage 2 applied |MAD-Z| > 3.5 within each group×speed cell to all 23 variables. Overall, 11 observations were removed in Stage 1 and 145 in Stage 2. QC was applied to all available observations within each group; only pairs with valid data for both members at a given variable and speed entered the linear mixed model (LMM). Available pairs per speed: 10 km/h n = 52, 11 km/h n = 50, 12 km/h n = 50, 13 km/h n = 49, 14 km/h n = 47, 15 km/h n = 45.
