# Supplementary material for: The Biomechanical Signature of Elite Distance Running: A Matched Pairs Analysis Across Multiple Speeds
Source: Scand J Med Sci Sports. 2026 Oct 21;36(11):e70385. doi: 10.1111/sms.70385 (PMC13687571; doi:10.1111/sms.70385)
Supplement: Supplementary file 2 [file SMS-36-e70385-s002.docx]

**Table S3.** Descriptive statistics and within-pair effect sizes for elite (ELT) and recreational (CON) runners across six running speeds (10–15 km/h) for all 23 biomechanical variables included in the primary analysis.

| Domain - Variable | Group | | 10 km/h  (n = 52) | | *d*_z_  [95% CI] | | 11 km/h  (n = 50) | | *d*_z_  [95% CI] | 12 km/h  (n = 50) | *d*_z_  [95% CI] | 13 km/h  (n = 49) | *d*_z_  [95% CI] | 14 km/h  (n = 47) | *d*_z_  [95% CI] | 15 km/h  (n = 45) | *d*_z_  [95% CI] |
| --- | --- | --- | --- | --- | --- | --- | --- | --- | --- | --- | --- | --- | --- | --- | --- | --- | --- |
| Spatiotemporal | |  | |  | |  | |  | | | | | | | | |  |
| Step Frequency (steps/min) | **ELT** | | 163.5 ± 6.2 | | +0.27  [−0.01; +0.56] | | 165.3 ± 6.2 | | +0.16 [−0.13; +0.44] | 166.6 ± 6.6 | +0.03  [−0.25; +0.31] | 168.1 ± 6.7 | −0.15  [−0.44; +0.14] | 169.9 ± 6.6 | −0.17  [−0.47; +0.12] | 172.2 ± 6.3 | −0.14  [−0.44; +0.16] |
|  | **CON** | | 161.1 ± 6.5 | |  |  | 163.9 ± 6.5 | |  | 166.4 ± 6.8 |  | 169.4 ± 8.1 |  | 171.6 ± 8.5 |  | 173.5 ± 7.9 |  |
| Step Length (m) | **ELT** | | 1.00 ± 0.10 | | +0.19  [−0.09; +0.48] | | 1.09 ± 0.09 | | +0.08  [−0.21; +0.36] | 1.19 ± 0.10 | +0.07 [−0.22; +0.36] | 1.28 ± 0.11 | +0.16 [−0.14; +0.45] | 1.38 ± 0.10 | +0.10  [−0.20; +0.40] | 1.47 ± 0.12 | +0.19  [−0.11; +0.49] |
|  | **CON** | | 0.98 ± 0.07 | |  |  | 1.09 ± 0.06 | |  | 1.19 ± 0.05 |  | 1.27 ± 0.06 |  | 1.36 ± 0.07 |  | 1.44 ± 0.10 |  |
| **Contact Time (ms)** | **ELT** | | 275 ± 15 | | **−0.73**  **[−1.04; −0.41]** | | 263 ± 15 | | **−0.63**  **[−0.94; −0.32]** | 252 ± 14 | **−0.64**  **[−0.95; −0.33]** | 241 ± 15 | **−0.56**  **[−0.86; −0.25]** | 232 ± 13 | **−0.61**  **[−0.92; −0.29]** | 222 ± 12 | **−0.53**  **[−0.84; −0.21]** |
|  | **CON** | | 296 ± 25 | |  |  | 278 ± 22 | |  | 267 ± 20 |  | 255 ± 19 |  | 245 ± 18 |  | 234 ± 17 |  |
| **Flight Time (ms)** | **ELT** | | 91 ± 20 | | **+0.49**  **[+0.19; +0.78]** | | 100 ± 17 | | **+0.41**  **[+0.10; +0.71]** | 110 ± 16 | **+0.54**  **[+0.24; +0.84]** | 116 ± 17 | **+0.54**  **[+0.22; +0.84]** | 121 ± 17 | **+0.58**  **[+0.26; +0.89]** | 124 ± 17 | **+0.51**  **[+0.18; +0.83]** |
|  | **CON** | | 75 ± 23 | |  |  | 88 ± 20 | |  | 94 ± 21 |  | 99 ± 22 |  | 104 ± 21 |  | 110 ± 21 |  |
| **Contact Ratio (%)** | **ELT** | | 74.7 ± 4.1 | | **−0.67**  **[−0.98; −0.36]** | | 72.4 ± 4.2 | | **−0.52**  **[−0.82; −0.22]** | 69.8 ± 4.1 | **−0.59**  **[−0.89; −0.29]** | 67.6 ± 4.1 | **−0.59**  **[−0.89; −0.27]** | 65.7 ± 3.7 | **−0.65**  **[−0.97; −0.33]** | 64.1 ± 3.4 | **−0.59**  **[−0.91; −0.27]** |
|  | **CON** | | 80.3 ± 6.6 | |  |  | 76.2 ± 5.4 | |  | 74.1 ± 5.3 |  | 72.0 ± 5.5 |  | 70.3 ± 5.3 |  | 67.7 ± 4.9 |  |
| **Flight Ratio (%)** | **ELT** | | 25.3 ± 4.1 | | **+0.66**  **[+0.35; +0.98]** | | 27.6 ± 4.2 | | **+0.52**  **[+0.22; +0.82]** | 30.1 ± 4.1 | **+0.59**  **[+0.29; +0.89]** | 32.3 ± 4.1 | **+0.59**  **[+0.27; +0.89]** | 34.1 ± 4.1 | **+0.65**  **[+0.33; +0.97]** | 36.0 ± 3.4 | **+0.59**  **[+0.27; +0.91]** |
|  | **CON** | | 20.0 ± 6.0 | |  |  | 23.8 ± 5.4 | |  | 25.9 ± 5.3 |  | 27.9 ± 5.4 |  | 29.8 ± 5.2 |  | 32.0 ± 5.1 |  |
| **Stride Angle (°)** | **ELT** | | 2.5 ± 0.9 | | **+0.42**  **[+0.13; +0.71]** | | 2.8 ± 0.9 | | **+0.47**  **[+0.17; +0.77]** | 2.9 ± 0.8 | **+0.52**  **[+0.22; +0.82]** | 3.1 ± 0.8 | **+0.58**  **[+0.27; +0.89]** | 3.1 ± 0.7 | **+0.63**  **[+0.31; +0.95]** | 3.1 ± 0.7 | **+0.52**  **[+0.20; +0.82]** |
|  | **CON** | | 1.9 ± 1.0 | |  |  | 2.1 ± 0.9 | |  | 2.2 ± 0.8 |  | 2.3 ± 0.9 |  | 2.3 ± 0.8 |  | 2.5 ± 0.8 |  |
| Stiffness & Force | |  | |  | |  | |  | | | | | | | | |  |
| **Leg Stiffness (kN/m)** | **ELT** | | 8.0 ± 1.1 | | **+0.52**  **[+0.22; +0.82]** | | 8.1 ± 1.1 | | **+0.56**  **[+0.25; +0.86]** | 7.9 ± 1.0 | **+0.59**  **[+0.28; +0.89]** | 8.0 ± 1.1 | **+0.57**  **[+0.25; +0.88]** | 7.9 ± 1.1 | **+0.62**  **[+0.30; +0.94]** | 7.8 ± 1.1 | **+0.51**  **[+0.19; +0.83]** |
|  | **CON** | | 7.2± 1.1 | |  |  | 7.2 ± 1.1 | |  | 7.1 ± 1.1 |  | 7.1 ± 1.2 |  | 7.0 ± 1.1 |  | 7.1 ± 1.0 |  |
| **Vertical Stiffness (kN/m)** | **ELT** | | 14.6 ± 1.0 | | **+0.32**  **[+0.02; +0.61]** | | 15.4 ± 1.0 | | **+0.40**  **[+0.10; +0.71]** | 16.1 ± 1.3 | **+0.43**  **[+0.13; +0.72]** | 16.8 ± 1.2 | **+0.26**  **[−0.03; +0.55]** | 17.6 ± 1.2 | **+0.34**  **[+0.04; +0.64]** | 18.7 ± 1.4 | **+0.26**  **[−0.04; +0.56]** |
|  | **CON** | | 14.2 ± 1.1 | |  |  | 14.8 ± 1.2 | |  | 15.5 ± 1.3 |  | 16.4 ± 1.6 |  | 17.0 ± 1.6 |  | 18.2 ± 1.8 |  |
| **Peak Vertical GRF (BW)** | **ELT** | | 3.4 ± 0.3 | | **+0.63**  **[+0.32; +0.94]** | | 3.6 ± 0.3 | | **+0.49**  **[+0.17; +0.80]** | 3.8 ± 0.3 | **+0.66**  **[+0.34; +0.97]** | 4.0 ± 0.3 | **+0.58**  **[+0.26; +0.89]** | 4.1 ± 0.4 | **+0.64**  **[+0.31; +0.96]** | 4.3 ± 0.4 | **+0.58**  **[+0.25; +0.90]** |
|  | **CON** | | 3.0 ± 0.4 | |  |  | 3.3 ± 0.4 | |  | 3.4 ± 0.4 |  | 3.6 ± 0.4 |  | 3.7 ± 0.4 |  | 3.9 ± 0.4 |  |
| **Vertical GRF Rate (BW/s)** | **ELT** | | 35.8 ± 2.1 | | **+0.75**  **[+0.43; +1.06]** | | 37.6 ± 2.0 | | **+0.67**  **[+0.35; +0.99]** | 39.3 ± 2.0 | **+0.71**  **[+0.40; +1.03]** | 40.8 ± 2.3 | **+0.58**  **[+0.27; +0.89]** | 42.3 ± 2.4 | **+0.59**  **[+0.27; +0.90]** | 44.1 ± 2.6 | **+0.48**  **[+0.17; +0.78]** |
|  | **CON** | | 33.3 ± 2.7 | |  |  | 35.5 ± 2.6 | |  | 37.1 ± 2.6 |  | 38.8 ± 2.7 |  | 40.3 ± 2.7 |  | 42.3 ± 2.7 |  |
| Horizontal GRF Rate (BW/s) | **ELT** | | 6.0 ± 0.5 | | +0.10  [−0.20; +0.39] | | 6.8 ± 0.5 | | −0.03  [−0.32; +0.27] | 7.5 ± 0.6 | −0.10  [−0.39; +0.19] | 8.2 ± 0.7 | −0.14  [−0.43; +0.16] | 9.1 ± 0.6 | −0.20  [−0.50; +0.10] | 10.0 ± 0.8 | −0.01  [−0.31; +0.30] |
|  | **CON** | | 6.0 ± 0.3 | |  |  | 6.8 ± 0.3 | |  | 7.6 ± 0.3 |  | 8.3 ± 0.4 |  | 9.2 ± 0.4 |  | 10.0 ± 0.5 |  |
| Total Force Rate (BW/s) | **ELT** | | 87.2 ± 5.0 | | +0.35  [+0.05; +0.64] | | 95.2 ± 5.0 | | +0.31  [+0.01; +0.61] | 101.5 ± 5.3 | +0.01  [−0.27; +0.30] | 108.1 ± 5.8 | −0.12  [−0.42; +0.17] | 115.9 ± 5.6 | −0.21  [−0.51; +0.10] | 126.0 ± 8.4 | −0.14  [−0.43; +0.16] |
|  | **CON** | | 84.5 ± 5.3 | |  |  | 93.0 ± 5.7 | |  | 101.3 ± 6.1 |  | 109.0 ± 6.3 |  | 117.4 ± 6.3 |  | 127.6 ± 10.1 |  |
| Impact & Shock | |  | |  | |  | |  | | | | | | | | |  |
| Shock (g) | **ELT** | | 8.5 ± 1.5 | | +0.01  [−0.27; +0.28] | | 8.9 ± 1.5 | | −0.17  [−0.45; +0.12] | 9.5 ± 1.6 | −0.11  [−0.40; +0.18] | 9.7 ± 1.4 | −0.28  [−0.58; +0.01] | 10.0 ± 1.5 | −0.25  [−0.54; +0.05] | 10.0 ± 1.5 | −0.39  [−0.70; −0.09] |
|  | **CON** | | 8.5 ± 1.7 | |  |  | 9.3 ± 1.5 | |  | 9.7 ± 1.3 |  | 10.2 ± 1.5 |  | 10.5 ± 1.5 |  | 10.8 ± 1.5 |  |
| Braking Gs (g) | **ELT** | | 4.9 ± 1.0 | | +0.17  [−0.11; +0.46] | | 5.3 ± 1.1 | | +0.13  [−0.16; +0.41] | 5.5 ± 1.2 | +0.10  [−0.19; +0.38] | 5.8 ± 1.2 | +0.16  [−0.13; +0.44] | 5.9 ± 1.2 | +0.10  [−0.19; +0.40] | 6.0 ± 1.2 | −0.01  [−0.30; +0.29] |
|  | **CON** | | 4.6 ± 1.1 | |  |  | 5.1 ± 1.0 | |  | 5.4 ± 1.0 |  | 5.6 ± 1.1 |  | 5.7 ± 1.1 |  | 6.0 ± 1.1 |  |
| Impact Gs (g) | **ELT** | | 6.6 ± 1.5 | | −0.10  [−0.38; +0.18] | | 7.0 ± 1.6 | | −0.24  [−0.53; +0.05] | 7.2 ± 1.6 | −0.30  [−0.59; −0.01] | 7.5 ± 1.7 | −0.34  [−0.63; −0.05] | 7.6 ± 1.6 | −0.43  [−0.73; −0.13] | 7.6 ± 1.8 | −0.40  [−0.70; −0.09] |
|  | **CON** | | 6.8 ± 1.7 | |  |  | 7.5 ± 1.6 | |  | 7.9 ± 1.6 |  | 8.3 ± 1.6 |  | 8.5 ± 1.6 |  | 8.6 ± 1.7 |  |
| Medio-lateral Gs (g) | **ELT** | | 0.50 ± 0.23 | | +0.06  [−0.21; +0.34] | | 0.59 ± 0.27 | | +0.11  [−0.18; +0.40] | 0.58 ± 0.23 | −0.18  [−0.46; +0.10] | 0.59 ± 0.28 | −0.30  [−0.59; −0.00] | 0.58 ± 0.28 | −0.23  [−0.53; +0.07] | 0.61 ± 0.31 | −0.28  [−0.58; +0.02] |
|  | **CON** | | 0.48 ± 0.24 | |  |  | 0.56 ± 0.24 | |  | 0.65 ± 0.30 |  | 0.71 ± 0.34 |  | 0.69 ± 0.33 |  | 0.78 ± 0.47 |  |
| Foot Kinematics | |  | |  | |  | |  | | | | | | | | |  |
| **Footstrike Type (a.u.)** | **ELT** | | 11.9 ± 3.2 | | **+0.60**  **[+0.29; +0.90]** | | 11.6 ± 3.4 | | **+0.63  [+0.32; +0.94]** | 11.5 ± 3.4 | **+0.67  [+0.36; +0.98]** | 11.4 ± 3.5 | **+0.65  [+0.33; +0.96]** | 11.3 ± 3.6 | **+0.66**  **[+0.34; +0.98]** | 11.2 ± 3.7 | **+0.58**  **[+0.26; +0.89]** |
|  | **CON** | | 9.2 ± 3.3 | |  |  | 8.6 ± 3.2 | |  | 8.5 ± 3.2 |  | 8.5 ± 3.2 |  | 8.4 ± 3.1 |  | 8.3 ± 3.1 |  |
| Pronation Excursion (°) | **ELT** | | 15.4 ± 3.7 | | +0.33  [+0.04; +0.62] | | 16.9 ± 3.8 | | +0.43  [+0.13; +0.74] | 17.5 ± 4.4 | +0.40  [+0.10; +0.70] | 18.6 ± 4.5 | +0.41  [+0.10; +0.71] | 19.8 ± 4.8 | +0.45  [+0.14; +0.76] | 21.5 ± 4.9 | +0.46  [+0.14; +0.77] |
|  | **CON** | | 13.4 ± 4.3 | |  |  | 14.0 ± 4.7 | |  | 14.8 ± 4.9 |  | 15.6 ± 5.2 |  | 16.4 ± 5.6 |  | 17.9 ± 5.0 |  |
| Max Pronation Velocity (°/s) | **ELT** | | 604 ± 168 | | +0.10  [−0.18; +0.38] | | 661 ± 185 | | +0.14  [−0.14; +0.43] | 706 ± 197 | +0.12  [−0.16; +0.40] | 765 ± 213 | +0.13  [−0.16; +0.42] | 819 ± 235 | +0.10  [−0.19; +0.39] | 867 ± 247 | +0.05  [−0.24; +0.34] |
|  | **CON** | | 581 ± 157 | |  |  | 627 ± 155 | |  | 677 ± 161 |  | 734 ± 181 |  | 792 ± 195 |  | 853 ± 201 |  |
| **Swing Excursion (°)** | **ELT** | | 85.3 ± 8.8 | | **−0.26**  **[−0.54; +0.02]** | | 92.9 ± 8.9 | | **−0.33**  **[−0.61; −0.03]** | 99.3 ± 9.4 | **−0.35**  **[−0.64; −0.05]** | 105.0 ± 9.6 | **−0.27**  **[−0.56; +0.02]** | 109.8 ± 9.8 | **−0.29**  **[−0.58; +0.01]** | 115.2 ± 10.5 | **−0.30**  **[−0.60; +0.00]** |
|  | **CON** | | 88.7 ± 9.3 | |  |  | 97.2 ± 9.4 | |  | 103.7 ± 8.9 |  | 108.9 ± 10.9 |  | 114.1 ± 11.1 |  | 120.0 ± 12.0 |  |
| Vertical Dynamics |  | |  | |  | |  | |  |  |  |  |  |  |  |  |  |
| Vertical Oscillation (cm) | **ELT** | | 9.3 ± 1.2 | | −0.16  [−0.44; +0.12] | | 9.0 ± 1.2 | | −0.15  [−0.44; +0.13] | 8.7 ± 1.2 | +0.05  [−0.24; +0.34] | 8.5 ± 1.2 | +0.12  [−0.17; +0.41] | 8.2 ± 1.2 | +0.20  [−0.10; +0.50] | 7.6 ± 1.1 | +0.07  [−0.24; +0.39] |
|  | **CON** | | 9.6 ± 1.4 | |  |  | 9.2 ± 1.2 | |  | 8.6 ± 1.4 |  | 8.3 ± 1.4 |  | 7.8 ± 1.6 |  | 7.5 ± 1.4 |  |
| **Vertical Speed (m/s)** | **ELT** | | -1.84 ± 0.31 | | **−0.19**  **[−0.48; +0.09]** | | -1.94 ± 0.26 | | **−0.23**  **[−0.51; +0.06]** | -2.10 ± 0.36 | **−0.34**  **[−0.63; −0.05]** | -2.22 ± 0.38 | **−0.41**  **[−0.71; −0.11]** | -2.32 ± 0.38 | **−0.43**  **[−0.73; −0.13]** | -2.43 ± 0.41 | **−0.38**  **[−0.68; −0.07]** |
|  | **CON** | | -1.75 ± 0.29 | |  |  | -1.84 ± 0.31 | |  | -1.92 ± 0.34 |  | -1.99 ± 0.36 |  | -2.08 ± 0.37 |  | -2.21 ± 0.40 |  |

*Values are mean ± SD. d_z_ [95% CI] is Cohen's d_z_ calculated from within-pair differences at each speed (ELT − CON; mean difference divided by the SD of the differences), with 95% confidence intervals derived from the non-central t distribution. Bold variable names indicate a FDR-significant group main effect (q < 0.05) in the linear mixed model. ELT = elite runners; CON = recreational runners; GRF = ground reaction force; BW = body weight; a.u. = arbitrary units.*
