# Supplementary material for: The Biomechanical Signature of Elite Distance Running: A Matched Pairs Analysis Across Multiple Speeds
Source: Scand J Med Sci Sports. 2026 Oct 21;36(11):e70385. doi: 10.1111/sms.70385 (PMC13687571; doi:10.1111/sms.70385)
Supplement: Supplementary file 3 [file SMS-36-e70385-s003.docx]

Table S2. Anthropometric characteristics of elite (ELT) and recreational (CON) runners before and after 1:1 nearest-neighbour matching.

| Variable | ELT | CON | SMD | p | ΔSMD |
| --- | --- | --- | --- | --- | --- |
| Before matching (ELT n = 55; CON pool n = 117) | | | | | |
| Sex (% male) | 80.0% (44/55) | 70.1% (82/117) | +0.23 | 0.199ᵃ |  |
| Age (years) | 27.3 ± 6.5 | 33.5 ± 10.0 | −0.74 | <0.001 |  |
| Height (cm) | 175.1 ± 8.5 | 174.9 ± 9.2 | +0.03 | 0.871 |  |
| Body mass (kg) | 64.0 ± 9.3 | 71.9 ± 12.4 | −0.71 | <0.001 |  |
| BMI (kg/m2) | 20.8 ± 1.8 | 23.4 ± 3.1 | −1.03 | <0.001 |  |
| After matching (ELT n = 52; CON n = 52) | | | | | |
| Sex (% male) | 78.8% (41/52) | 78.8% (41/52) | 0.00 | 1.000ᵃ | **−0.23** |
| Age (years) | 27.3 ± 6.4 | 31.6 ± 10.7 | −0.49 | 0.002 | **−0.25** |
| Height (cm) | 175.8 ± 8.4 | 175.2 ± 8.4 | +0.07 | 0.229 | **+0.04** |
| Body mass (kg) | 64.7 ± 9.2 | 67.1 ± 8.9 | −0.27 | <0.001 | **−0.44** |
| BMI (kg/m2) | 20.8 ± 1.8 | 21.8 ± 1.8 | −0.53 | <0.001 | **−0.50** |

Values are mean ± SD for continuous variables and percentage (n) for sex. SMD: standardised mean difference; for continuous variables d = (mean₁ − mean₂) / pooled SD; for binary variables d = (p₁ − p₂) / √[(p₁(1−p₁) + p₂(1−p₂)) / 2]. Negative SMD indicates ELT < CON. ΔSMD: change in SMD from before to after matching. Pre-matching p-values from independent-samples t-test; post-matching p-values from paired t-test on within-pair differences; ᵃ Fisher's exact test. Body mass and age were retained as covariates in the LMM to account for residual within-pair imbalance.
